# Accuracy of Self-Report and Pill-Count Measures of Adherence in the FEM-PrEP Clinical Trial: Implications for Future HIV-Prevention Trials

**DOI:** 10.1007/s10461-014-0859-z

**Published:** 2014-08-07

**Authors:** Kawango Agot, Douglas Taylor, Amy L. Corneli, Meng Wang, Julie Ambia, Angela D. M. Kashuba, Caleb Parker, Ansley Lemons, Mookho Malahleha, Johan Lombaard, Lut Van Damme

**Affiliations:** 1 Impact Research and Development Organization, P.O. Box 9171-40141, Kisumu, Kenya; 2 FHI 360, Durham, NC USA; 3 The Eshelman School of Pharmacy, University of North Carolina, Chapel Hill, NC USA; 4 Setshaba Research Centre, Soshanguve, Pretoria, South Africa; 5 JOSHA Research, Bloemfontein, South Africa; 6 Present Address: KAVI-Institute of Clinical Research, University of Nairobi, Nairobi, Kenya; 7 Present Address: The Bill & Melinda Gates Foundation, Seattle, WA USA

**Keywords:** PrEP, Adherence, Positive predictive value, Self-report, Pill-count

## Abstract

Oral emtricitabine/tenofovir disoproxil fumarate (FTC/TDF) has been evaluated as pre-exposure prophylaxis (PrEP). We describe the accuracy of self-reported adherence to FTC/TDF and pill counts when compared to drug concentrations in the FEM-PrEP trial. Using drug concentrations of plasma tenofovir (TFV) and intracellular tenofovir diphosphate (TFVdp) among a random sub-sample of 150 participants assigned to FTC/TDF, we estimated the positive predictive value (PPV) of four adherence measures. We also assessed factors associated with misreporting of adherence using multiple drug-concentration thresholds and explored pill use and misreporting using semi-structured interviews (SSIs). Reporting use of ≥1 pill in the previous 7 days had the highest PPV, while pill-count data consistent with missing ≤1 day had the lowest PPV. However, all four measures demonstrated poor PPV. Reported use of oral contraceptives (OR 2.26; *p* = 0.014) and weeks of time in the study (OR 1.02; *p* < 0.001) were significantly associated with misreporting adherence. Although most SSI participants said they did not misreport adherence, participant-dependent adherence measures were clearly unreliable in the FEM-PrEP trial. Pharmacokinetic monitoring remains the measure of choice until more reliable participant-dependent measures are developed.

## Introduction

In the past decade, oral tenofovir disoproxil fumarate (TDF) with or without emtricitabine (FTC) has been evaluated in clinical trials as pre-exposure prophylaxis (PrEP) for its safety and efficacy in preventing HIV acquisition [1–7]. Several studies have demonstrated that PrEP is efficacious and that higher adherence levels lead to higher levels of effectiveness [2, 4, 5, 8]. In contrast, the FEM-PrEP and Vaginal and Oral Interventions to Control the Epidemic (VOICE) trials did not show any effect of oral TDF, oral FTC/TDF, or vaginal TDF gel on HIV acquisition in women. Despite excellent self-reported adherence by participants, investigators from both trials cited low actual adherence as the likely reason for the lack of efficacy [3, 6].

Methods for measuring adherence in clinical trials often include participant self-report, electronic monitoring devices, pill counts, pharmacy refills, and drug concentrations in biological samples. Each measure has its own strengths and weaknesses [9]. Data from participant self-report (the most common adherence measure) are subject to bias, as their accuracy depends on the participants’ memory or comfort in providing truthful responses. Biomarkers, such as drug concentrations in plasma, can be costly to implement. Further, plasma concentrations may only provide information about the last few days of drug dosing and may be subject to “white-coat adherence” bias, whereby a participant only takes the drug shortly before a study visit to give the appearance of adhering. Other biological measures, such as intracellular levels in peripheral blood mononuclear cells or red blood cells or measures of drug concentrations in hair samples, may provide a more comprehensive understanding of longitudinal adherence.

FEM-PrEP was a phase III, randomized, double-blind, placebo-controlled trial to assess the efficacy and safety of once-daily FTC/TDF in reducing HIV acquisition among women who were at higher risk of HIV infection [3]. Here, we use drug concentration data on plasma tenofovir (TFV) and intracellular tenofovir diphosphate (TFVdp) to assess the accuracy of three self-report measures and one pill-count measure of adherence from the FEM-PrEP clinical trial. We also describe qualitative data on non-adherence, the misreporting of adherence, and participants’ comfort level in reporting occurrences of non-adherence.

## Methods

### Ethics Statement

All associated ethics and regulatory committees approved the trial. All trial participants provided written informed consent prior to their participation.

### Study Overview

Details of the FEM-PrEP trial have been reported elsewhere [3]. Briefly, a total of 2,120 participants were enrolled at four African sites: Bondo, Kenya; Pretoria and Bloemfontein, South Africa; and Arusha, Tanzania. Participants received client-centered adherence counselling every 4 weeks for up to 52 weeks [10]. Adherence counselling was provided by trained counsellors; participants’ answers to the quantitative study-product adherence questionnaire were not reviewed by counselors prior to counseling. Pharmacists provided limited pill-taking messages when dispensing pill bottles.

### Data Collection

#### Quantitative Participant Self-Report

Toward the beginning of every visit and prior to counseling on adherence, local FEM-PrEP study interviewers administered a quantitative study-product adherence questionnaire at the study clinic; counselors did not administer the questionnaire. During this face-to-face interview, participants were asked the number of days they took the study pill in the past 7 days and how often (never, rarely/almost never, sometimes, usually/almost usually, or always) they took the study pill in the past 4 weeks. Various techniques thought to improve the reliability of participants’ responses were used, such as using a shorter time interval for recalling the number of pills taken; using an estimation question for longer time intervals; and letting participants know at the beginning of the interview that study staff members understand that some participants may adhere and some may not, to make clear that perfect adherence is not expected [9]. Interviewers were also trained to assure participants that they would not be upset with reports of non-adherence, to not express an opinion on the responses received, and to provide no counseling in response to reported non-adherence.

#### Pill Counts

Each participant was given a bottle containing 30 pills at visits scheduled for every 28 days (−4/+2 days). Staff members were allowed to re-dispense up to seven previously returned pills; thus, a participant would take home between 30 and 37 pills. At all visits, information was obtained on the number of pills returned and dispensed. The difference between the number of pills received at the previous visit and the number of pills returned represented the number of pills assumed used by pill count. This number was compared with the number of days that had elapsed between the previous visit and the current visit.

#### Plasma and Intracellular Drug Concentrations

Among a random sub-cohort of 150 participants assigned to FTC/TDF (50 participants from each site where HIV infections occurred: Bondo, Bloemfontein, and Pretoria), we assessed stored plasma and upper layer of packed cells (ULPCs) for TFV and FTC, and TFVdp and FTC-triphosphate (FTCtp) drug concentrations, respectively, at each follow-up visit. Details of the laboratory methods are described elsewhere [11]. Briefly, analytes were measured in plasma or ULPCs using protein precipitation followed by liquid chromatography with tandem mass spectrometry. All calibrators and quality control samples were within 15 % of the nominal value for both within-day and between-day runs.

#### Qualitative Interviews

We conducted SSIs with three groups of trial participants to explore study pill use, misreporting of self-reported adherence, and comfort level in reporting non-adherence. The first group consisted of participants who seroconverted at the Bondo and Pretoria sites and who returned for their study visits. These interviews were conducted at weeks 1, 4, and 8 post-HIV diagnosis; questions on adherence were asked at weeks 1 and 4. The second group consisted of HIV-negative participants who were interviewed during the trial (referred to as the “HIV-negative group”). These interviews were conducted every 3–4 months among a 5 % random sample at the Bondo and Pretoria sites. We explored different adherence-related topics during these interviews using a standard set of questions. The adherence topics varied at different time points, according to our ongoing assessments of what information would be most useful to inform trial implementation at the time. The questions, however, remained the same whenever the topic was explored in an interview. The third group was a random sample of HIV-negative trial participants who completed at least one remaining study visit after the decision was made to halt the study early because of lack of effectiveness. These exit interviews were conducted at the Bondo, Pretoria, and Bloemfontein sites. Each group included participants who were randomized to receive FTC/TDF or placebo.

All SSIs were conducted at the study clinic by local members of the FEM-PrEP study staff. Interviewers were different staff members from those who conducted the quantitative study-product adherence questionnaire, and they were not involved in any clinical or adherence-related activities. Participants in all three groups were asked to describe the context of any days in which they were unable to take the study pill. Participants were also asked if they ever misreported adherence to the study staff and, if so, the reasons why; participants in the HIV-negative group were asked these questions only at specific time points. During the second year of trial implementation, we also asked participants in the HIV-negative group to describe their comfort level in reporting instances of non-adherence to study staff members. The purpose of these questions was to identify situations in which participants were unable to take the study pills regularly, possible patterns of adherence, and reasons for misreporting pill use in order to enhance adherence counseling and reporting; our goal was not to triangulate the qualitative and quantitative adherence data because the SSIs did not focus on a specific time point. Interviews were audiotaped when participants gave permission. Expanded notes were taken for participants who declined to be audiotaped.

### Adherence Measures and Analysis Methods

We assessed the accuracy of four self-report and pill-count adherence measures by estimating their PPV—the percentage of study visits where the adherence level indicated by the measure was supported by drug-concentration data. If the applicable drug concentration threshold was not achieved, then we considered the participant to have misreported her adherence (i.e., the probability of misreport for a particular measure equals 1 minus the PPV of the measure). Appropriate drug thresholds for each measure were selected based on TFV/FTC pharmacokinetic data in plasma [11, 12] and previously determined TFVdp and FTCtp concentrations in the ULPC matrix under steady-state and single-dose conditions [12]. We used plasma TFV concentrations (with a half-life of approximately 9 h) to assess the accuracy of self-reported pill use in the 7 days prior to specimen collection, and a composite measure of TFV in plasma and TFVdp in ULPCs (with a half-life of >72 h) to assess the accuracy of self-report and pill-count data over 4 week intervals. The four adherence measures were as follows.

#### Measure #1

Participant self-reports of taking pills on at least 6 of the 7 days prior to specimen collection, which would be consistent with very high or perfect adherence in the previous week. For this adherence level, a participant would have to have taken a pill in the 24–48 h prior to the clinic visit, in which case the TFV plasma level would be ≥10 ng/mL.

#### Measure #2

We were also interested in knowing whether participants might report “any” pill taking more accurately than “high” or “perfect” adherence. Hence, in the second measure, we assessed participant self-reports of taking at least one pill in the 7 days prior to specimen collection. In this case, a participant’s TFV level in plasma is expected to be ≥0.25 ng/mL.

#### Measure #3

The third measure consisted of pill counts consistent with the participant missing no more than 1 day of pill use during the entire 4 week visit interval—which would correspond to near-perfect adherence. For this adherence level, the plasma TFV concentration is expected to be ≥10 ng/mL and the concentration of TFVdp in ULPCs is expected to be ≥100,000 fmoles/mL. If the plasma TFV concentration was <10 ng/mL, then the participant was unlikely to have taken a pill in the previous 2 days. If her TFVdp concentration was <100,000 fmoles/mL, then she was unlikely to have taken pills consistently in the first few weeks of the study interval.

#### Measure #4

The last measure consisted of participant self-reports that they “usually” or “always” took the pills in the previous 4 weeks. For purposes of this analysis, we interpreted “usually or always” to mean taking at least five pills per week. As for measure #3, a participant’s plasma TFV concentration is expected to be ≥10 ng/mL and her concentration of TFVdp in ULPCs is expected to be ≥100,000 fmoles/mL if she usually or always took the pills.

The PPV of each measure was summarized overall and by week of follow-up, after excluding: (1) intervals when participants did not have sufficient product to cover the visit interval (participants were not asked to report their adherence if they missed their previously scheduled supply visit and therefore could not have adhered as per protocol); and (2) intervals that were <10 days (in which case TFVdp concentrations might not reflect adherence during the interval due to the long half-life of the metabolized drug).

We used logistic regression to assess associations between misreporting adherence and three sets of factors: those assessed at baseline, those assessed repeatedly during trial participation, and those assessed when participants exited the study. Generalized estimating equation methods with robust variance estimation were used to account for repeated measures on participants. Bivariate analyses were conducted first, followed by a multivariate analysis that initially included all bivariate factors with *p* < 0.10, and then backwards elimination to obtain a final model with factors significant at the 0.05 level.

We evaluated baseline factors of site, age, education, marital status, parity, use of highly effective contraception at screening [oral contraceptives (OCs), intrauterine devices, implants, injectable contraceptives, or female sterilization], use of OCs at enrollment, and having a sexually transmitted infection (gonorrhea, chlamydia, syphilis, or trichomoniasis). We also assessed time-dependent factors: types of partners in the previous 4 weeks (primary partner only versus more than one sexual partner); having sex without a condom; having reported a gastrointestinal event (nausea, vomiting, or diarrhea) any time prior to the visit; and HIV risk perception (dichotomized as none versus small, moderate or high chance due to the low frequency of responses in each of the small, moderate and high categories). Factors we assessed from the participants’ last adherence questionnaire included beliefs about their randomly assigned treatment arm (placebo, FTC/TDF, or “don’t know”) and how much participants liked taking the daily pill. We made an a priori decision to use measure #1 (reports of using pills in 6 of the previous 7 days) for the primary analysis of factors associated with the misreport of adherence, but similar models were used to conduct sensitivity analyses based on each remaining measure.

Applied thematic analysis was used to analyze the qualitative data [13]. Structural codes related to non-adherence, reporting missed pills, and comfort level in reporting adherence were applied to the interview text by two analysts using NVivo 9 [14]. The analysts independently coded the same samples of randomly selected transcripts, reviewed codes, resolved differences, modified the codebook, and re-coded as needed to ensure inter-coder reliability. Coding reports were reviewed to identify themes, which were subsequently confirmed by three analysts. Summary reports were written to describe themes and to list frequencies and illustrative quotes. For the interviews with participants who seroconverted, we included data only from participants whose clinical data contributed to the primary analysis of the trial.

## Results

The random sub-cohort of 150 participants assigned to FTC/TDF were scheduled to make 1,364 visits prior to study closure, out of which 1,172 (86 %) contributed to analyses based on TFV concentrations alone (missed study visits and protocol-defined product withdrawals accounted for nearly all excluded data). A further 11 visits had insufficient specimens for ULPC assessment, leaving 1,161 records for analyses based on both TFV and TFVdp concentrations.

### Pill Counts and Self-Reported Adherence

The pill-count data indicated that the participants missed no more than 1 day of pill-taking during 82 % of eligible study intervals. Participants reported taking pills on at least 1 of the previous 7 days at nearly all visits (99.6 %) and on at least 6 of the previous 7 days at 94.7 % of visits. They also reported usually or always taking pills in the previous 4 weeks at 99.1 % of visit intervals.

**Fig. 1:**
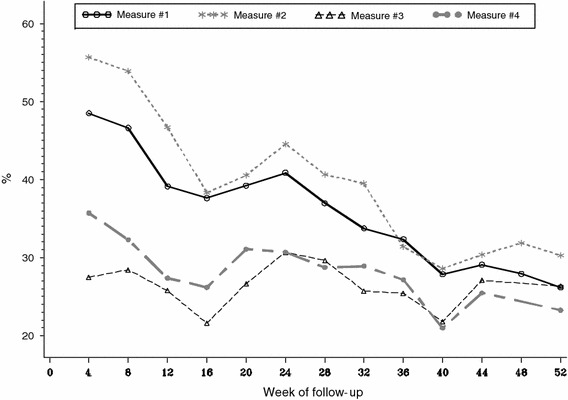
Positive predictive value (the percent of visits where drug concentration data were consistent with the reported adherence level) over time. Refer to section on “Adherence Measures and Analysis Methods” for a detailed description of each measure

The PPVs of each measure are summarized in Table 1 for all visits combined, with PPV plotted over time in Fig. 1. Measure #2 (reports of using at least one pill in the previous 7 days) had the highest PPV, followed by Measure #1 (reports of using pills on at least 6 of the previous 7 days), Measure #4 (reports of usually or always taking pills) and Measure #3 (pill-count data consistent with missing no more than 1 day during the interval). Each of the four measures demonstrated a poor PPV at week 4 (27–56 %), which generally decreased over time (23–30 % by week 52).

**Table 1:** Positive predictive values of each adherence measure averaged over time

Adherence measure	PPV (%)	n/N
Self-reported pill use in previous 7 days		
Measure #1: ≥10 ng/mL plasma TFV among visits where participants report ≥6 days taking pills	38.0	420/1,105
Measure #2: ≥0.25 ng/mL plasma TFV among visits where participants report ≥1 days taking pills	42.2	490/1,162
Pill counts during each visit interval		
Measure #3: ≥10 ng/mL plasma TFV and ≥100,000 fmoles TFVdp/mL in ULPCs among visits where pill-count data indicate no more than 1 day without pill use	26.2	249/952
Self-reported pill use in previous 4 weeks		
Measure #4: ≥10 ng/mL plasma TFV and ≥100,000 femtomoles TFVdp/mL in ULPCs among visits where participants report usually or always taking pills	28.7	329/1,146

### Factors Associated with Misreporting Adherence

In bivariate analyses, time in the study (OR 1.02 per week of follow-up; *p* = 0.002) and choice of OCs to fulfill the study’s requirement for the use of a contraceptive method at enrollment (OR 2.13; *p* = 0.021) were each associated with increased odds of misreporting adherence by Measure #1 (Table 2). Having previously been pregnant (OR 0.57; *p* = 0.084) and considering oneself to be at some risk of HIV (OR 0.62; *p* = 0.057) were not significant but met our pre-specified criteria for possible inclusion in an adjusted, multivariate model. In the final model, only OC use at enrollment (OR 2.26; *p* = 0.014) and time in the study (OR 1.02 per week of follow-up; *p* < 0.001) remained significant. These two factors were also significantly associated with the misreporting of adherence in sensitivity analyses using each of the other self-reported measures and the pill-count measure. However, considering oneself to be at risk of HIV (*p* = 0.041) and not liking daily pill taking (*p* = 0.026) were also associated with less misreporting for Measure #2, and study site was associated with misreporting for Measure #4 (*p* = 0.047, with participants from Bloemfontein less likely to misreport; results not shown).

**Table 2:** Odds ratios (OR) for associations with misreporting of adherence

Factor (“yes” vs. “no” unless specified)	Bivariate		Multivariate^a^	
	OR (95 % CI)	*p* value	OR (95 % CI)	*p* value
Baseline variables				
Study site^b^		0.164		
Pretoria, South Africa	1.87 (0.96, 3.62)			
Bondo, Kenya	1.86 (0.89, 3.90)			
Age^c^		0.465		
25-29	0.68 (0.35, 1.32)			
≥30	0.72 (0.31, 1.67)			
≥10 years of education	1.27 (0.66, 2.43)	0.478		
Married	1.19 (0.61, 2.29)	0.610		
Using OCs at enrollment	2.13 (1.12, 4.05)	0.021	2.26 (1.18, 4.35)	0.014
Using highly effective contraception at screening	1.43 (0.79, 2.59)	0.241		
Ever pregnant before enrollment	0.57 (0.30, 1.08)	0.084		
Diagnosed with STI at baseline	1.24 (0.67, 2.29)	0.498		
Variables assessed at study exit				
Like daily pill-taking	0.55 (0.25, 1.18)	0.124		
Randomization arm belief (placebo vs. Truvada or don’t know)	1.48 (0.46, 4.77)	0.507		
Time-dependent variables^d^				
Unprotected sex	1.24 (0.77, 1.98)	0.378		
Have other sexual partners	1.07 (0.51, 2.24)	0.851		
Gastrointestinal event	0.48 (0.15, 1.56)	0.225		
HIV risk perception^e^	0.62 (0.38, 1.01)	0.057		
Time in study (weeks)	1.02 (1.01, 1.03)	0.002	1.02 (1.01, 1.03)	<0.001

Less than 10 ng/mL plasma TFV when participants reported taking pills at least 6 of the previous 7 days

^a^Includes factors that remained significant at the 0.05 level in final adjusted model

^b^Bloemfontein, South Africa, as reference

^c^Age 18–24 as reference

^d^Measured at nearest visit on or before assessment of drug concentration

^e^Some chance (small, moderate, high) versus no chance of getting HIV

### Semi-Structured Interviews

Fifty-six women who seroconverted were included in the initial sample. Two of these participants were removed; one described non-adherence because of an investigator-initiated product interruption (hence reasons for non-adherence were not participant related), and another participated only in the week 8 interview (where no questions on adherence were asked). This reduced our total sample size to 54 (29 were randomized to FTC/TDF and 25 to placebo) and all discussed pill use and misreporting. Among the 180 participants in the HIV-negative group, 176 participants (FTC/TDF = 84, placebo = 92) discussed pill use, 63 (FTC/TDF = 31, placebo = 32) described misreporting, and 48 (FTC/TDF = 24, placebo = 24) explained their comfort level in reporting occurrences of non-adherence to study staff members. Among the 57 exit interviews conducted, 56 participants (FTC/TDF = 31, placebo = 25) described pill use and 54 (FTC/TDF = 29, placebo = 25) described misreporting.

#### Study Product Non-use

Among the HIV-negative SSI group that was interviewed during the clinical trial, many participants (56 %; n = 99: FTD/TDF = 44, placebo = 55) said that they had never missed taking their study pill. A common response was:

*I do not miss. I take it every day.*—a 24-year-old single woman from Pretoria who had 11 years of education

However, some participants in all three SSIs groups said that there were instances when they had not taken the study pill. Some of these participants said that they had missed their daily pill only once. Others reported that they had missed pills over several consecutive days. Such accounts of product non-use were more common among participants in the exit SSI group (75 %; n = 42: FTC/TDF = 24, placebo = 18) and the seroconverter SSI group (59 %; n = 32: FTC/TDF = 17, placebo = 15) than among participants in the HIV-negative SSI group (44 %; n = 77: FTC/TDF = 40, placebo = 37). Regardless of the type of group, descriptions of non-adherence suggested that participants missed pills only occasionally, primarily because of short-term travel or forgetfulness, and that this non-adherence was not likely reflective of their overall adherence:

*Sometimes I would forget [to take the study pill] as I left home thinking I would come back, [but] I didn’t. That is when I would forget to drink them, but when I was home I was able to drink them every day. [Interviewer: So, how often would you say that happened?] It didn’t happen much, maybe two or three times.*—a 19-year-old single woman from Pretoria who had 11 years of education and was in the HIV-negative SSI group

#### Misreporting Product Non-use

Among the 54 participants who seroconverted, only two (4 %, both FTC/TDF) said they had ever over-reported pill use to study staff members; all others stated that they had either given accurate reports or never missed taking a pill.

*There are some two days that I forgot to take. I was not around and I did not carry the pills. But I came [to the study clinic] and reported.*—a 20-year-old married woman from Bondo who had 7 years of education

Similarly, a minority of participants (19 %; n = 10: FTC/TDF = 7, placebo = 3) in the exit interviews and very few participants (5 %; n = 3: FTC/TDF = 1, placebo = 2) in the HIV-negative group said they had inaccurately reported missing pills to study staff members during the trial. Of those who said they did not always report missing pills, fears of being scolded or of being dropped from the study were cited as key reasons.

#### Comfort with Reporting Non-adherence

Most of the 48 participants in the HIV-negative group (90 %; n = 43: FTC/TDF = 20, placebo = 23) said they would be comfortable reporting occurrences of non-adherence.

*I am comfortable. I would just inform them so that they will know*—a 20-year-old single woman from Bondo who had 10 years of education

Only five participants (10 %: FTC/TDF = 4, placebo = 1) said they were uncomfortable telling staff that they did not take their study pills as instructed.

## Discussion

In the FEM-PrEP trial, misreporting of adherence was very common—averaged over time, the positive predictive values were less than 45 % for each of the four adherence measures we assessed. Reports of taking at least one pill in the previous 7 days appeared to have a somewhat higher predictive value than the other measures. However, accurate reports of this adherence level may not be especially meaningful in terms of understanding the effectiveness of a product intended to be used every day. More telling, self-reports and pill counts that were intended to capture consistent pill use over 4 week periods had the lowest predictive values (less than 30 % on average).

We had a large sample of drug concentrations for analysis (nearly 1,200 longitudinal visit records from 150 participants). However, our data on drug concentrations in plasma TFV and intracellular TFVdp could be misleading in certain circumstances. For example, different underlying patterns of adherence can result in similar drug concentrations when the latter are assessed infrequently. In contrast, similar patterns of adherence can lead to different drug concentrations due to heterogeneity of pharmacokinetic processes (e.g. absorption and metabolism) across participants or populations. Likewise, unknown drug–drug interactions could lead to bias when classifying adherence. Recognizing these limitations, we chose conservative drug concentration thresholds—ones that should be achieved with a reasonable degree of certainty if a participant adhered at the reported level—to assess the potential accuracy of adherence reports. As a consequence, we may have over-estimated the PPV, and under-estimated misreporting, of the assessed adherence measures. We also relied on plasma concentrations when assessing reports of high adherence in the previous 7 days (Measure #1). Due to the short half-life of TFV in plasma, a participant who only took a pill shortly before going to the clinic (“white coat adherence”) could achieve plasma concentrations similar to those of participants who truly adhered at a high level. We would have misclassified such women as correctly reporting high adherence, and under-estimated the rate of misreporting. Finally, we recognize that the data obtained from the SSIs did not focus on a specific time point, so we cannot triangulate the qualitative and quantitative results on adherence.

In the primary multivariate analysis, the choice of OCs for pregnancy prevention at enrollment was significantly associated with misreporting. Given that women choosing to use OCs also had very high pregnancy rates [15], this result suggests that participants may have concealed instances of non-adherence to both contraceptive use and study pill use. Time in the study also remained significant in the primary multivariate analysis, with the level of misreporting generally increasing over time. This may reflect a trend towards lower adherence over time rather than an indication that the participants increased the rate at which they reported adhering.

Given the magnitude of over-reporting in quantitative self-reported measures, we believe that participants also over-reported adherence through the qualitative interviews. One of the strengths of qualitative methods is the ability to explore personal or sensitive information [16, 17]. However, the participants’ fears of perceived repercussions of non-adherence (such as being discontinued from the trial) may not necessarily be alleviated based on the type of method (quantitative or qualitative) used to solicit experiences with adherence when the information is ultimately reported by participants to study staff members at the study clinic. Although there were slight differences in the participants’ qualitative descriptions of non-adherence, of their misreporting of adherence, and of their comfort level in reporting occurrences of non-adherence between the two study arms, the overall number of participants is too small to make any valid conclusions about these differences. Our findings from the exit interviews also suggest that participants may have been more comfortable sharing accounts of product non-use after the trial announced it was closing early. Based on a similar premise, we conducted follow-up interviews with FEM-PrEP participants to identify the reasons for non-adherence and misreporting many months after all FEM-PrEP clinical and community activities were completed. The findings will be presented elsewhere.

With such high levels of misreporting, we must not only continue to improve methods to reduce socially desirable responses through participant self-report, but also closely examine the reasons why participants join HIV-prevention clinical trials in the first place. Improvements to adherence counselling and participant self-reports will only be beneficial if study populations enroll in such trials with some interest in taking the study product. Given that many FEM-PrEP participants said that they adhered to pill taking as instructed, it is difficult to fully understand why they did not in fact take the study product yet consistently came for clinic visits and underwent the study procedures, or why they concealed their non-adherence. Clearly, participants perceived some benefit of remaining in the clinical trial while not taking the study product. Other FEM-PrEP data have suggested that several participants took part in the FEM-PrEP trial for the personal benefits they would receive, such as care and treatment for common illnesses and ongoing HIV testing [18]. Further research is needed in this area.

The large discrepancy we found between adherence assessed through self-report or pill-count and adherence assessed through drug concentrations or other biomarkers is not unique to FEM-PrEP. In the Carraguard trial [19], self-reported adherence was 94 %, yet on the basis of applicator testing, the study gel was estimated to have been used in only 42.1 % of sex acts. In the HSV-2 suppression therapy trial [20], acyclovir was detected in 55 % of participants’ urine, yet adherence by pill count was 90 %. Adherence measured by pill count and self-report in the iPrEx trial [21] was 93 %, yet a sub-study of drug concentrations showed that only 50 % of participants were actually swallowing their pills and only 9 % of those who seroconverted (n = 36) had any drug in their cells. Furthermore, in the VOICE trial [6], adherence to the pills or microbicide was 93 %, according to product counts and self-reports; however, only 28–29 % of participants assigned to TDF or FTC/TDF, and 22 % of participants assigned to the TFV 1 % gel, had detectable drug levels in blood (or vaginal fluids in the case of the microbicide).

## Conclusions

In summary, participant-dependent measures of adherence were unreliable in FEM-PrEP. Although expensive and logistically challenging, pharmacokinetic monitoring remains the measure of choice until more reliable participant-dependent measures are developed. Methods are currently available to measure drug concentrations in participants’ blood, cells, or other biological matrices, such as cervicovaginal fluids, in trials with topical products. In future clinical trials, data on drug concentrations could be utilized in addition to more reliable participant-dependent measures and pharmacy refills to inform overall adherence on a real-time basis. Such results, however, should not be used to counsel individual participants as that may un-blind the study; rather, the results should be used to offer generalized counselling to all participants about the overall level of adherence in the study. In addition, future research should explore whether in-depth interviews conducted by non-clinic-based staff members and at locations outside the study clinic allow participants who are experiencing problems with adherence to be more willing to disclose their difficulties or concerns.
